# Effect of disease duration on foveal microvasculature assessed by OCTA in type 2 diabetes mellitus without clinical diabetic retinopathy

**DOI:** 10.1186/s40942-025-00694-1

**Published:** 2025-06-15

**Authors:** David Leonardo Cruvinel Isaac, Alexandre Caiado Ferreira Pires, Laís Lauria Neves, Jamil Miguel Neto, Heitor do Amaral Simões, Karime Fugihara Iwamoto, Raphael Toledo Remiggi, Leticia Pinheiro de Freitas, Alexandre Chater Taleb, Marcos Avila

**Affiliations:** 1 Department of Ophthalmology, Federal University of Goias, Goiania, Brazil; 2 Hospital de Olhos Aparecida, Goiania, Brazil; 3 Centro de Referencia em Oftalmologia da Universidade Federal de Goias, Avenida, numero 741, cep, Goiania, 74605-020 Goias Brazil

**Keywords:** Optical coherence tomography angiography, Foveal avascular zone, Vessel density, Diabetic retinopathy, Type 2 diabetes

## Abstract

**Background:**

The objective of this study was to establish a comparison between the vessel density (VD) and foveal avascular zone (FAZ) of patients with type 2 diabetes mellitus (T2DM) who lacked clinical signs of diabetic retinopathy (DR) and non-diabetic patients using optical coherence tomography angiography (OCTA).

**Methods:**

A cross-sectional comparative case-control study (unpaired) was carried out at two tertiary hospitals. All subjects underwent optical coherence tomography angiography (OCTA) examination (DRI OCT Triton Swept Source, Topcon, Japan). The average VD in the superficial capillary plexus (SCP) and the deep capillary plexus (DCP), the FAZ area (mm2) in SCP, and DCP were taken into analysis. The time since the diagnosis of T2DM was used to stratify patients with diabetes between 5 and 10 years and those with a diagnosis of more than 10 years.

**Results:**

Compared to non-diabetic controls, the parafoveal VD in both SCP and DCP was significantly reduced in the eyes of T2DM patients without clinical DR (*p* < 0.001). Additionally, the VD was also statistically reduced in T2DM diagnosed more than 10 years ago compared to T2DM cases diagnosed between 5 and 10 years ago (*p* < 0.001). The FAZ area in both plexuses was larger in T2DM eyes compared to controls (*p* < 0.001). The FAZ area was enlarged in DCP (*p* = 0.04), but there was no significance of FAZ area in SCP when comparing patients with T2DM diagnosed between 5 and 10 years ago to those diagnosed more than 10 years ago (*p* = 0.06).

**Conclusion:**

In diabetic patients with long-term diagnosed disease, OCTA was shown to be capable of detecting preclinical microvascular foveal abnormalities prior to the development of clinically apparent retinopathy. According to our findings, OCTA has the potential to be a promising instrument for the early detection of vascular micro-abnormalities and the routine screening of diabetic eyes.

## Background

The global prevalence of diabetes mellitus (DM) is increasing exponentially each year due to increased life expectancy and changes in dietary habits. In 2010, it affected approximately 285 million people (6.4% of the world’s population), and it is estimated that by 2030, approximately 439 million people (7.7% of the world’s population) will have the disease [1].

Diabetic retinopathy (DR) is a microangiopathy secondary to DM [2] and the leading cause of visual impairment in working-age adults in the western population. It is characterized by peripheral and central capillary ischemia, macular hyperpermeability, and a secondary increase in angiogenic factors. Diabetic macular edema (DME) is the consequence of the internal blood-retinal barrier’s impairment, which results in the extravasation of fluid from the retinal vessels into the retina. Imaging or fundoscopic evaluation is used to diagnose DME when retinal thickening affects the macula [3].

With the development of optical coherence tomography angiography (OCTA), which is performed without intravenous contrast, it is possible to evaluate both the superficial and deep retinal vascular plexuses as well as the choroidal vessels through the flow of red blood cells by means of multiple slices (scans). In combination with OCTA, *en face* optical coherence tomography (OCT) allows the segmentation of the retinal layers and provides a frontal scan to determine the affected area among the different planes of the retina [4].

Some capillarity changes can be detected on the OCTA before any clinical manifestation of DR. It is known that DR causes a loss of capillaries which can lead to an enlargement of foveal avascular zone (FAZ) and a reduced vessel density (VD). The OCTA is a non-invasive and rapid method to analyze both parameters [5]. The changes found in OCTA can add important information about the structure of the retina in diabetic patients. It may be beneficial for ophthalmologists to gain a more comprehensive understanding of the progression of this disease, which would enable them to identify microvascular changes prior to the detection of diabetic retinopathy during a clinical examination.

The purpose of this study was to investigate the retinal microvascular differences by measuring VD and FAZ and comparing between diabetic eyes with no evidence of DR on fundoscopy and control eyes of patients without DM using a Swept-Source (SS) OCTA (DRI OCT Triton Swept Source, Topcon, Japan).

## Methods

This was a cross-sectional comparative case-control study (unpaired), carried out at CEROF-UFG (Centro de Referencia em Oftalmologia da Universidade Federal de Goias) and the OCTA and structural OCT examinations were performed at HOA (Hospital de Olhos Aparecida), from January 10th to June 30th, 2022. This study adhered to the tenets of the Declaration of Helsinki and was approved by the institutional review boards of the participating institutions. All subjects voluntarily agreed to participate by signing the informed consent form.

### Inclusion criteria

Inclusion criteria for the case group were patients over 18 years of age, diagnosed with DM2 more than 5 years ago, and no signs of DR on funduscopic examination and color fundus photography. The inclusion criteria for the control group were patients over 18 years of age, with no previous diagnosis of DM, no other ocular disease, and best corrected visual acuity better than 20/30. The date of diagnosis of T2DM was obtained from the patients’ medical records or by self-report of how long they had known about the disease.

### Exclusion criteria

Exclusion criteria were patients with signs of DR (such as microaneurysms, microhemorrhages, exudates, intraretinal microvascular abnormalities - IRMAs, neovascularization); any pathology that could interfere with the test results, such as cataract (lens opacities preventing adequate imaging); corneal opacity (such as leukoma or keratopathies); myopia with spherical equivalent greater than 6 diopters, best corrected visual acuity ≤ 20/40; previous macular pathology (such as macular hole, epiretinal membrane, age-related macular disease, vascular occlusions, and retinal dystrophies); patients without laboratory tests to assess DM in the last six months, OCTA scans of unsatisfactory quality, such as those that produced images of inadequate quality with an index of less than 40, patients who underwent cataract surgery in the last three months, and patients who did not know how long they had been diagnosed with the disease.

### Ophthalmic evaluation

The patients included in the study were examined by two Board certified ophthalmologists by the Brazilian Council of Ophthalmology and Brazilian Medical Association. They underwent a complete questionnaire, anamnesis, past pathological history, ophthalmological history, dynamic refraction to obtain the best corrected visual acuity, using a manual phoropter and Snellen chart. Decimal visual acuity was converted to a logarithmic scale with the smallest angle of resolution (logMAR). Ophthalmic examinations were performed in the following order: anterior segment biomicroscopy; Goldmann applanation tonometry; retinal mapping (RM) with an indirect binocular ophthalmoscope and a 20D lens, under dilated pupils. After RM, patients were referred for color retinography, which was performed by a single trained, masked technician using the Canon cx-1 retinal camera (Canon, Tokyo, Japan). Color fundus photographs were used by both examiners to identify possible signs of retinopathy, not seen on funduscopic examination. After the examination at CEROF-UFG, patients who were eligible for structural OCT and OCTA according to the inclusion and exclusion criteria were scheduled to undergo the examinations at another hospital (maximum interval of 15 days from the first examination). At the HOA, patients underwent OCT and OCTA in a dark room without the need for eye drops for pupil dilation by an experienced and masked technician using the DRI OCT Triton (DRI OCT Triton Swept Source, Topcon, Japan). T2DM patients were further stratified into patients diagnosed between 5 and 10 years and patients diagnosed for more than 10 years.

### Assessment of foveal avascular zone and vessel density

The VD and FAZ area were assessed with HD 3 × 3 mm OCTA images with four repeated scans centered on the fovea. Good image quality, according to the manufacturer of the OCT used in this study, is greater than 40. The automatic segmentation of the Triton device extends in the SCP from 2.6 μm below the internal limiting membrane and the lower limit of 15.6 μm below the inner plexiform layer (IPL)/inner nuclear layer (INL) interface; in the DCP, the upper limit of 15.6 μm below the IPL/INL interface and the lower limit of 70.2 μm below the IPL/INL interface.

After the examinations, the images were quantified, evaluated, and compared with the VD and FAZ area of the superficial and deep retinal plexuses using the software ImageJ. The analysis of VD and FAZ area at the SCP and DCP levels was performed with the *en face* image and image quality greater than 40 points (0-100) of the OCTA. Patients’ refraction correction was performed when analyzing images with ImageJ.

**Fig. 1:**
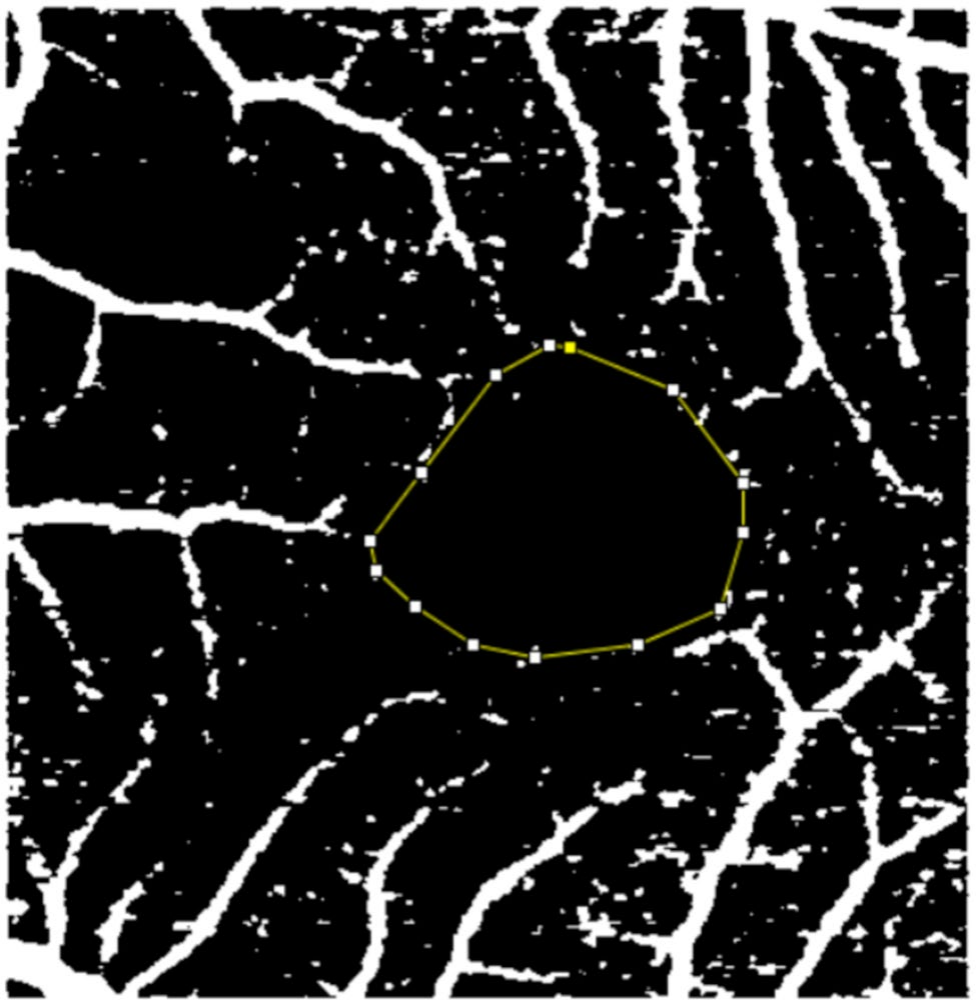
Points to define the area of the SCP of the FAZ in the ImageJ

**Fig. 2:**
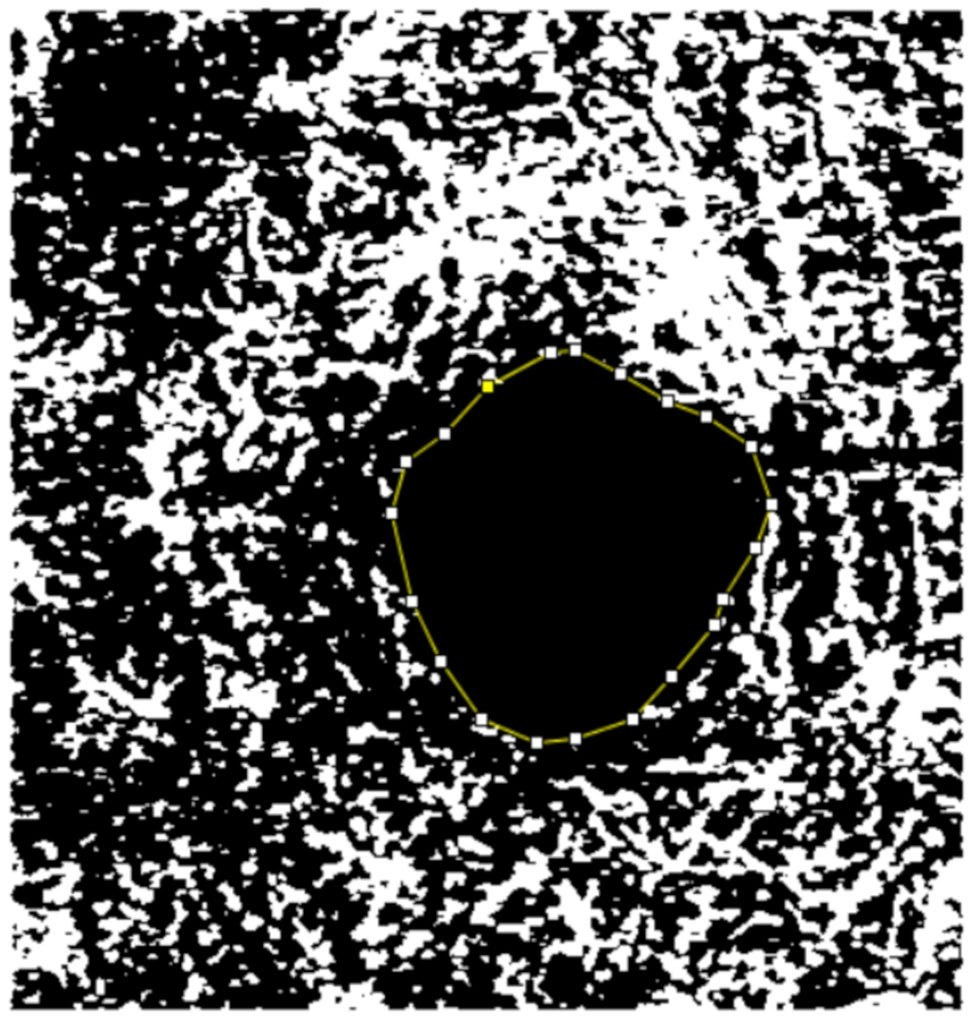
Points to define the area of the DCP of the FAZ in ImageJ

Images were exported from OCTA in PDF format. The projections were opened using the ImageJ image analysis program, version 1.49, which is in the public domain and available from the US National Institute of Health (Bethesda, USA. Available at: https://imagej.nih.gov/ij/). The 3 × 3 mm scans were converted to pixelated and binarized images. To assess the area of the superficial and deep plexus FAZ, the points of the avascular area were manually marked by two unmasked examiners at different times. The area contained within the geometric shape was automatically calculated by ImageJ in mm² according to the total number of pixels within the region, with the final value calculated as the average of the two measurements obtained (Figs. 1 and 2).

VD was expressed as a ratio by taking the total area of the vessel divided by the total area of the region analyzed over the entire 3 × 3 mm scan. After extracting the original images from the visualization software, the images were imported into ImageJ. A binarized image with thresholding, as implemented in the software, was used to measure VD. The application assumes that the image contains two classes of pixels that follow a bimodal distribution. It calculates the optimal threshold by minimizing the intra-class variance and maximizing the inter-class variance. The total number of pixels occupied by vessels was then divided by the total number of pixels in the entire image and the value was expressed as a ratio.

### Statistical analysis

The profile of patients in the control and T2DM groups was characterized using absolute frequency, relative frequency for categorical variables, and mean and standard deviation for continuous variables. The distribution of the profile between groups was tested using Pearson’s chi-squared test. The normality of the data was tested using the Kolmogorov-Smirnov test, while the comparison of FAZ area and VD values between groups was performed using analysis of variance (ANOVA) followed by Tukey’s post hoc test. Data were analyzed using the Statistical Package for Social Science (IBM Corporation, Armonk, USA) version 26.0, with a significance level of 5% (*p* < 0.05).

## Results

Initially, 96 eyes were considered for inclusion, of which 10 were excluded after meeting the exclusion criteria. A total of 86 eyes of 43 patients were included in the study. The case group included 52 eyes of 26 patients (12 male and 14 female) with T2DM, 30 eyes of 15 patients diagnosed between five and ten years ago and showing no signs of DR on fundus examination, and 22 eyes of 11 patients diagnosed with T2DM for more than ten years and showing no signs of DR. The control group consisted of 34 eyes of 17 patients (6 male and 11 female) with no previous diagnosis of T2DM.

The mean ± standard deviation (SD) age was 65.7 ± 10.6 years in the control group, 62.5 ± 10.1 years in the case group diagnosed 5–10 years ago and 65.4 ± 10.9 years in the case group diagnosed more than 10 years ago. No statistically significant difference was observed for age and eye laterality between cases and controls (*p* = 0.43 and *p* = 1.00, respectively) (Table 1).

**Table 1:** Profile characterization of patients in control and diabetes groups

	Groups			Total	*p*-value
	Control	DM 5 to 10 years	DM > 10 years		
Age (years)					
Mean value ± SD	65.7 ± 10.6	62.5 ± 10.1	65.4 ± 10.9	64.5 ± 10.5	0.43**
Age group *n* (%)					
43 to 59 years	10 (29.4)	10 (33.3)	8 (36.4)	28 (32.6)	0.85*
60 to 83 years	24 (70.6)	20 (66.7)	14 (63.6)	58 (67.4)	
Eye *n* (%)					
Right	17 (50.0)	15 (50.0)	11 (50.0)	43 (50.0)	1.00*
Left	17 (50.0)	15 (50.0)	11 (50.0)	43 (50.0)	

*Chi-square; **ANOVA; n, absolute frequency; %, relative frequency; SD, standard deviation

Parafoveal VD in both SCP and DCP was reduced in T2DM eyes without clinical DR compared to non-diabetic controls (*p* < 0.001), and the FAZ area in both plexuses increased in case eyes compared to controls (*p* < 0.001), as shown in Table 2.

**Table 2:** Results of comparison of FAZ and VD in control and DM groups without DR

	Control	DM > 5 years	*p*-value
FAZ ***SCP*** (mm^2^)	0.24 ± 0.04	0.37 ± 0.64	< 0.001*
VD ***SCP*** (%)	62.38 ± 0.87	56.52 ± 3.99	< 0.001*
FAZ ***DCP*** (mm^2^)	0.36 ± 0.03	0.47 ± 0.75	< 0.001*
VD ***DCP*** (%)	73.67 ± 1.12	65.32 ± 5.19	< 0.001*

ANOVA*

FAZ: foveal avascular zone; SCP: superficial capillary plexus; DCP: deep capillary plexus

VD: vascular density

When comparing VD results in SCP and DCP of controls to groups of T2DM subjects diagnosed between 5 and 10 years ago and more than 10 years ago, statistical significance was also observed (*p* < 0.001 and *p* < 0.001, respectively). The comparison of T2DM cases diagnosed between 5 and 10 years ago to those diagnosed more than 10 years ago was significant, showing reduced VD results (in SCP and DCP) in the group with longer duration of diabetes (*p* < 0.001). There was significant difference in DCP FAZ area (*p* = 0.04) and no significant difference of FAZ area in SCP when comparing cases diagnosed between 5 and 10 years ago to those diagnosed more than 10 years ago (*p* = 0.06), Table 3.

**Table 3:** Results of FAZ and VD comparisons of all groups

	Groups			*p* ^a^	*p* ^b^	*p* ^c^
	Control	DM 5 to 10 years	DM > 10 years			
FAZ/***SCP*** (mm^2^)	0.24 ± 0.04	0.36 ± 0.06	0.38 ± 0.07	< 0.001*	< 0.001*	0.06*
VD/***SCP*** (%)	62.38 ± 0.87	59.03 ± 1.48	53.73 ± 1.20	< 0.001*	< 0.001*	< 0.001*
FAZ/***DCP*** (mm^2^)	0.36 ± 0.03	0.45 ± 0.06	0.49 ± 0.09	< 0.001*	< 0.001*	0.04*
VD/***DCP*** (%)	73.67 ± 1.12	68.98 ± 1.54	61.67 ± 1.31	< 0.001*	< 0.001*	< 0.001*

ANOVA*

^a^Control vs. DM 5 to 10 years; ^b^Control vs. DM > 10 years; ^c^DM 5 to 10 years vs. DM > 10 years

FAZ: foveal avascular zone; SCP: superficial capillary plexus; DCP: deep capillary plexus

VD: vascular density; DM: diabetes mellitus

**Fig. 3:**
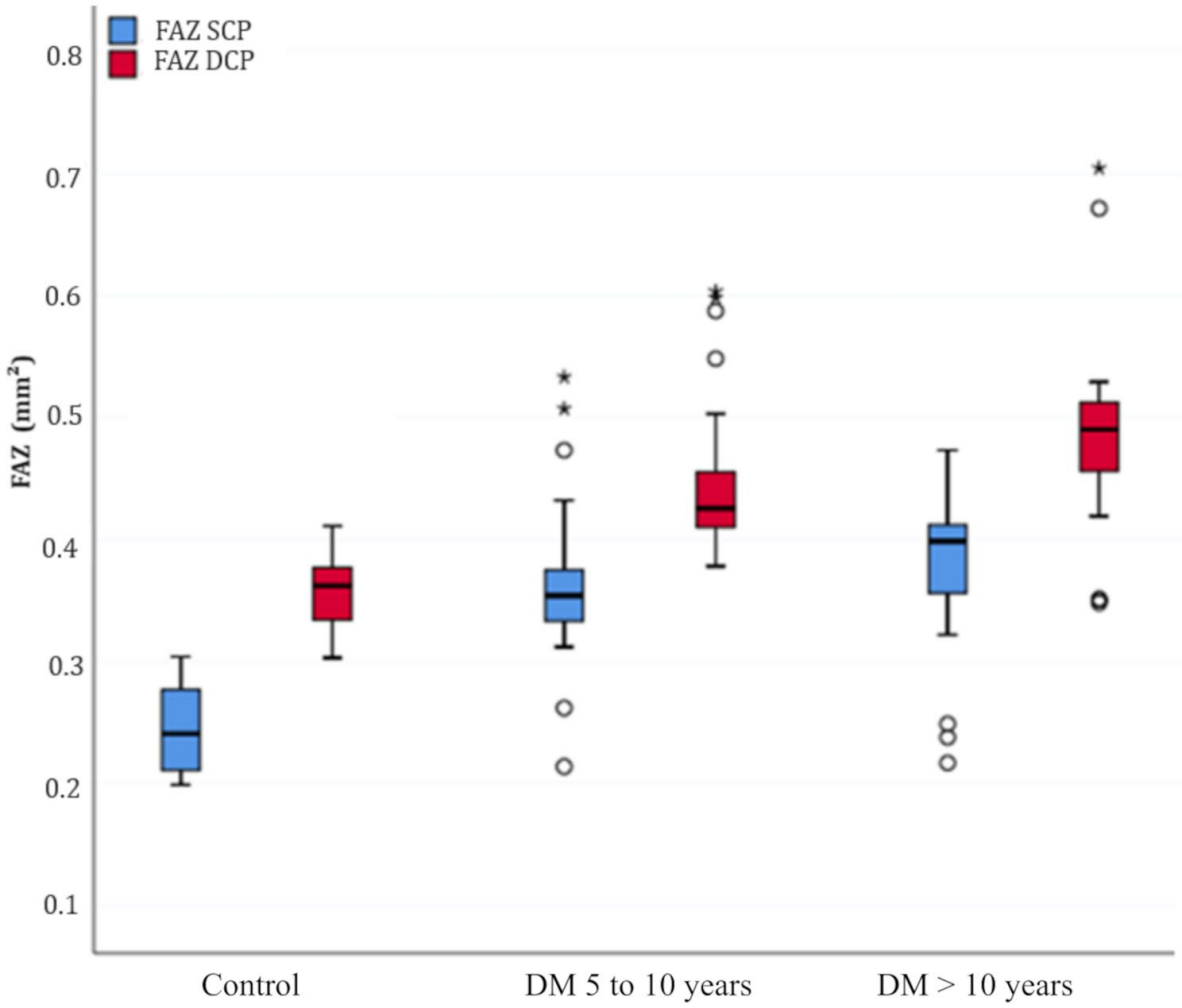
Boxplot graph shows the central tendency, symmetry, and dispersion of the FAZ values in mm^2^

**Fig. 4:**
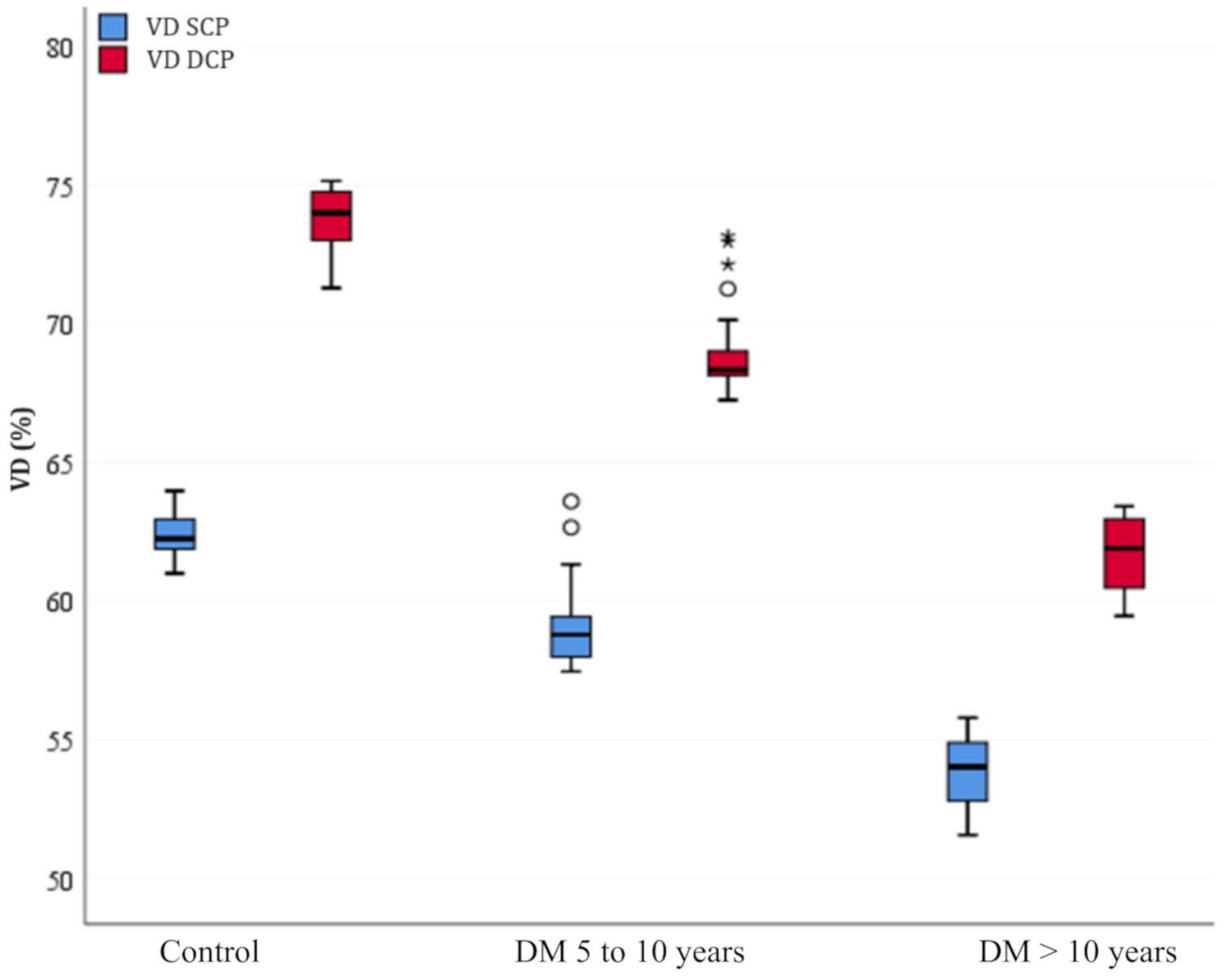
Boxplot graph showing the central tendency, symmetry, and dispersion of VD values in %

Figure 3 demonstrates a progressive increase in the FAZ area according to the time of disease, as well as Fig. 4 shows a reduction in VD with the time of disease.

## Discussion

The aim of the present study was to establish a comparison between the vessel density (VD) and foveal avascular zone (FAZ) of patients with type 2 diabetes mellitus (T2DM) who lacked clinical signs of diabetic retinopathy (DR) and non-diabetic patients using optical coherence tomography angiography (OCTA). This comparison would address the potential use of optical coherence tomography angiography as a method for screening and detecting eventual preclinical retinal changes in patients with T2DM with no signs of fundoscopic or retinography, based on foveal microvasculature evaluation. The identification of foveal microvascular lesions may highlight the necessity for more stringent ophthalmological monitoring and an invasive examination, such as fluorescein angiography.

One of the characteristics of DR is the progressive nature of the changes, from deep to superficial areas, although literature has not been able to validate a single pathophysiological mechanism that explains the chronology of the changes. Although Choi et al. initially showed a reduction in VD in SCP [7] many studies suggest that changes in the retinal circulation first originate in the outer layers, only to manifest later with the funduscopic findings of DR [8, 9, 10, 11 and 12]. The findings of Iwase et al. (2016) support this reasoning, as they showed a higher oxygen demand in the deeper retinal plexuses, justifying the onset of changes in this region [13]. As this was a cross-sectional analysis of the data, it was not possible to assess the chronology of VD changes, as both plexuses showed a reduction in patients in the case group (DM with more than 5 years of disease diagnosis).

There are some inconsistencies in the literature regarding the changes found in the FAZ. A study by Carnevali et al. using OCTA with ED technology (CIRRUS HD-OCT model 5000, Carl Zeiss Meditec, Jena, Germany) compared 25 patients diagnosed with type 1 DM with more than five years of disease and no DR findings on fundus examination with 25 patients in the control group without DM. It showed that there was no increase in the FAZ in the superficial and deep plexuses in the different groups [14]. Their results were contrary to the findings of the present study, which showed an increase in the FAZ area in patients in the case group (T2DM > 5 years of diagnosis without signs of DR) compared to the control group. A likely explanation is that the present study evaluated patients with T2DM, which is known to be a silent disease in its early stages, and therefore it is difficult to determine the real time of exposure to the disease [15]. Another factor that may have contributed to these findings is that Swept Source OCT has greater penetration than Spectral Domain OCTA, which would allow for more detailed visualization of the deep retinal plexus [16].

Another point to emphasize when interpreting the results is the fact that not finding an increase in FAZ area does not mean a complete absence of changes. For example, the study by Inanc et al. showed that the irregularity of the FAZ is part of the pathophysiological mechanism of the disease and that it occurs before the increase in its area [17], unlike the present study, which used a cross-sectional approach and evaluated only the area of the FAZ. Cao et al. used a methodology like the present study, but with limitations that should be considered. Although they also evaluated patients with T2DM and no signs of DR on fundus examination, they found no difference in SCP FAZ area between the groups analyzed (T2DM vs. control without DM) [18]. However, their study used the section size of 6 × 6 mm while in the present study the section size used was 3 × 3 mm, which may present better image quality of the fovea microvasculature, as shown by Ho et al. [19].

Other studies have shown increased FAZ in people with DM without signs of DR [20, 21]. In a study of 55 T2DM patients without signs of DR and 48 non-diabetics with an average disease duration of 17.9 years, significant differences in FAZ area of the SCP were found. The FAZ area of the DCP in the DM2 group was (0.37 ± 0.13 mm2) compared to the control group (0.29 ± 0.11 mm2; *P* = 0.012) [6]. Another study showed that in DM patients without evidence of DR, VD decreased in the superficial and deep vascular plexus, while ZAF increased in the superficial vascular plexus, suggesting that retinal vascular changes precede retinal structural changes [22], which is consistent with the hypothesis of the present study, although the patients were not followed longitudinally.

Even in the present study, although the aim was not to follow patients longitudinally, when comparing groups with different exposure times (T2DM 5–10 years vs. T2 DM > 10 years), an increase in FAZ was seen only in the deep plexus (*p* = 0.04), highlighting the importance of exposure time to the disease and that the involvement may begin in the deep layers. Therefore, longitudinal studies with longer exposure to T2DM are needed to better understand the progression of changes to the more superficial layers. However, it seems clear from this study that patients with longer duration of disease (T2DM > 10 years) tend to have a smaller VD and a larger FAZ than patients with T2DM 5–10 years, and these in turn have a larger FAZ and a smaller VD than patients without diagnosed DM and without retinal disease.

Considering both parameters, VD and ZAF, this study found similar results in literature. Onishi et al. investigated changes in the middle vascular plexus found a correlation between decreased vessel density in the middle and deep plexuses and the progression of diabetic retinopathy (DR), while no such correlation was observed with superficial plexus changes. Unfortunately, our study did not quantify the middle plexus, but this could be an interesting direction for future research [23].

In the present study, T2DM patients without signs of DR showed an increase in the FAZ in the superficial and deep plexus. In addition, other studies have shown an increased FAZ in individuals without signs of DR (preclinical changes), demonstrating that there may be changes in T2DM [20, 21, 22]. These studies support the findings of the present study, which showed an increase in FAZ in diabetic patients without evidence of DR. In this context, we can conclude the importance of OCTA in the evaluation of diabetic patients, as it is a non-invasive imaging exam and can show changes that cannot be detected by clinical examination.

This study has several limitations. The Self-reported DM duration is an important methodological limitation, since patients with type 2 diabetes mellitus may not have an exact determination of the duration of the disease. Patient evaluation and screening for inclusion were determined by clinical examination by two unmasked examiners, which may have introduced some error and bias. Fluorescein angiography was not performed. Patients with mild signs of DR who were classified as without retinopathy may have been included. However, not performing the exam was based on the premise that OCTA could be performed as an additional and non-invasive imaging exam to the clinical evaluation. The OCTA exam could, as it did, identify preclinical micro-abnormalities, signaling the need for additional exams such as fluorescent retinography itself or the need for more frequent clinical evaluation.

Another flaw was the size of the sample analyzed: 52 eyes (26 patients) in the case group and 34 (17 patients) eyes in the control group. The number of patients included was obtained consecutively during the stipulated period, and there was no statistical calculation of what the ideal sample size would be.

Still from the same perspective of limitation, this is a cross-sectional study with images taken at a single point in time, making it clear that a follow-up with longitudinal data from patients with DR could be even more useful to determine the role of OCTA in the assessment of VD and FAZ area in patients with DM. Finally, artifacts may affect the assessment of VD and FAZ, especially with DCP.

In the present study, we emphasized the presence of preclinical changes detected by OCTA in relation to FAZ area and VD in SCP and DCP, which precede any apparent abnormalities on clinical examination of the retina. These findings are relevant because they identify retinal microvascular changes in diabetic eyes before fundoscopic signs, using a non-invasive and rapid test. Therefore, OCTA may become an important tool not only in ophthalmology, allowing the observation of preclinical lesions of diabetic retinopathy, but also in internal medicine, allowing the early detection of target organ damage and the possibility of clinical-medical adjustments of glycemic control to prevent further damage.

The relevance of these findings is that OCTA can detect early changes in the retinal vasculature of diabetic eyes before they are visible on fundoscopy. Screening DM patients with dilated pupil fundoscopy is important and will not replace this assessment. In fact, by using OCTA, one could better assess VD and FAZ and detect the onset of DR even if the patient has normal visual acuity and no symptoms. Individuals with DM with “preclinical” changes may be more likely to develop retinopathy and therefore could be examined more frequently than those without changes.

In conclusion, OCTA may become a screening test in the ophthalmologic routine of patients with DM, thus contributing to the early diagnosis of DR and consequently reducing late diagnosis and major complications such as blindness. However, longitudinal studies are needed to determine the real value of using this type of technology to establish the relationship between foveal microvascular findings of VD and FAZ area and the onset of clinically detectable DR.

## Conclusions

Comparing the group of healthy patients to patients with DM for over five years who did not exhibit signs of DR on clinical examination, we observed a decrease in the vessel density and an increase in the foveal avascular zone in eyes of T2DM patients. In addition, the vessel density in both plexuses was reduced in the group of diabetic patients with a diagnosis of T2DM for more than 10 years, as compared to patients with a disease age of 5–10 years. The present study suggests that OCT-A can detect preclinical microvascular foveal lesions in diabetic patients without clinical signs of retinopathy. However, additional research is required with a larger number of patients. These changes are more apparent in patients with diabetes that persists for a prolonged period.

## Data Availability

No datasets were generated or analysed during the current study.

## Abbreviations

ANOVA: Analysis of Variance
CBO: Brazilian Council of Ophthalmology
CEROF-UFG: Ophthalmology Reference Center (from Portuguese “Centro de Referencia em Oftalmologia da Universidade Federal de Goias”)
DCP: Deep Capillary Plexus
DM: Diabetes mellitus
DME: Diabetic Macular Edema
DR: Diabetic retinopathy
FA: Fluorescein angiography
FAZ: Foveal Avascular Zone
HOA: Eye Hospital of Aparecida (from Portuguese “Hospital de Olhos Aparecida”)
IPL: Inner Plexiform Layer
IRMAs: Intraretinal Microvascular Abnormalities
mm: Millimeters
OCT: Optical Coherence Tomography
OCTA: Optical Coherence Tomography Angiography
SCP: Superficial Capillary Plexus
SS: Swept Source
UFG: Federal University of Goias (from Portuguese “Universidade Federal de Goias”)
VD: Vessel Density
